# Synthetic and Structural Studies of Ethyl Zinc *β*-Amidoenoates and *β*-Ketoiminates

**DOI:** 10.3390/molecules26113165

**Published:** 2021-05-25

**Authors:** Malavika A. Bhide, Joe A. Manzi, Caroline E. Knapp, Claire J. Carmalt

**Affiliations:** Department of Chemistry, University College London, 20 Gordon Street, London WC1H 0AJ, UK; malavika.bhide.14@ucl.ac.uk (M.A.B.); j.manzi@alumni.ucl.ac.uk (J.A.M.); caroline.knapp@ucl.ac.uk (C.E.K.)

**Keywords:** precursor, dimerisation, materials, zinc oxide, chemical vapour deposition

## Abstract

A set of heteroleptic ethyl zinc *β*-amidoenoates (**1**, **2**) and *β*-ketoiminates (**3**) of the form [LZnEt]_2_ with varying steric bulk have been synthesised via the reaction of diethylzinc with *β*-aminoenoate ligands HL^1^ and HL^2^ and *β*-ketoimine HL^3^. These complexes have been characterised via ^1^H and ^13^C NMR, mass spectrometry and single-crystal X-ray diffraction, which unambiguously determined all three structures as dimeric species in the solid state. We observe the unusual dimerisation of **1** and **2** through coordination of the central zinc atom to the methine carbon of the second monomer, which gives these complexes high reactivity. The thermal properties of complex **3** are explored via thermal gravimetric analysis (TGA), to investigate their potential as single-source precursors to zinc oxide, which shows that **3** has a significantly lower decomposition temperature as compared to its bis-ligated counterpart [Zn(L^3^)_2_], which gives **3** promise as a single-source precursor to zinc oxide.

## 1. Introduction

Zinc oxide (ZnO) is widely used as a transparent conducting oxide (TCO) material. TCOs combine high electrical conductivity with optical transparency, making them an essential and industrially important class of material. Although TCOs such as indium-doped tin oxide (ITO) and fluorine-doped tin oxide (FTO) are the most industrially produced for electronic applications, the scarcity and subsequent rising cost of indium requires a shift towards TCO materials incorporating more abundant elements, such as ZnO [1,2].

Deposition of ZnO thin films has been undertaken via several methods such as sputtering [3], pulsed vapour deposition [4], spray pyrolysis [5] and chemical vapour deposition (CVD) [6,7,8]. The advantage of using solution-based methods such as aerosol-assisted CVD is that precursors only have to be soluble, which therefore allows for a greater range of precursors to ZnO. Diethylzinc is the most widely used precursor to ZnO thin films via vapour deposition methods [9,10]. An in situ reaction with an oxygen source, commonly methanol, produces highly conductive ZnO films with excellent transparency. The drawback of using diethylzinc is its highly pyrophoric nature, making its use and handling difficult, especially for scale-up processes. A shift towards single-source precursors (SSPs) for ZnO thin films has led to several complexes being reported in the literature, including zinc acetate [11], zinc *β*-diketonates [12], zinc *β*-ketoiminates [13,14,15], zinc *β*-amidoenoates [16], zinc oxanes [17] and zinc alkoxides [18], to name a few. These complexes are mostly homoleptic bis-ligated species, and offer the advantage of stability and ease of handling. Although they are still air- and moisture-sensitive, they are relatively stable compared to pyrophoric diethylzinc. This however comes at a cost—the thermal decomposition temperatures of these complexes tend to be higher, as well as having greater carbon contamination in the resultant deposits upon decomposition [19,20].

In this work, we explore the synthesis of a set of heteroleptic ethyl zinc *β*-amidoenoates and *β*-ketoiminates with a view to using them as SSPs to ZnO. We hypothesise that the heteroleptic nature of these complexes will allow for both the high reactivity from the ethyl group while also maintaining the stability offered from a chelating ligand.

## 2. Results and Discussion

**Figure 1:**
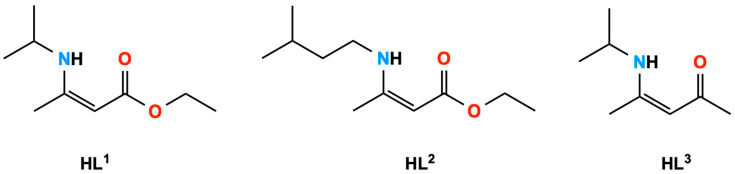
Ligands used in this work.

The *β*-aminoenoates ligands MeC(NH*^i^*Pr)CHC(O)OEt (HL^1^) and MeC(NH(CH_2_)_2_*^i^*Pr)CHC(O)OEt (HL^2^) were synthesised via 1:1 acid-catalysed condensation reactions between ethyl acetoacetate and isopropylamine and isopentylamine, respectively, using K-10 montmorillonite clay as the acid catalyst. The ligands were afforded in excellent yields using this method. Following standard synthetic routes for the preparation of *β*-ketoimine ligands, MeC(NH*^i^*Pr)CHC(O)Me (HL^3^) was synthesised via a simple 1:1 condensation reaction of acetylacetone and isopropylamine, and isolated as an orange oil of a high purity and with a good yield (Figure 1).

**Figure 2:**
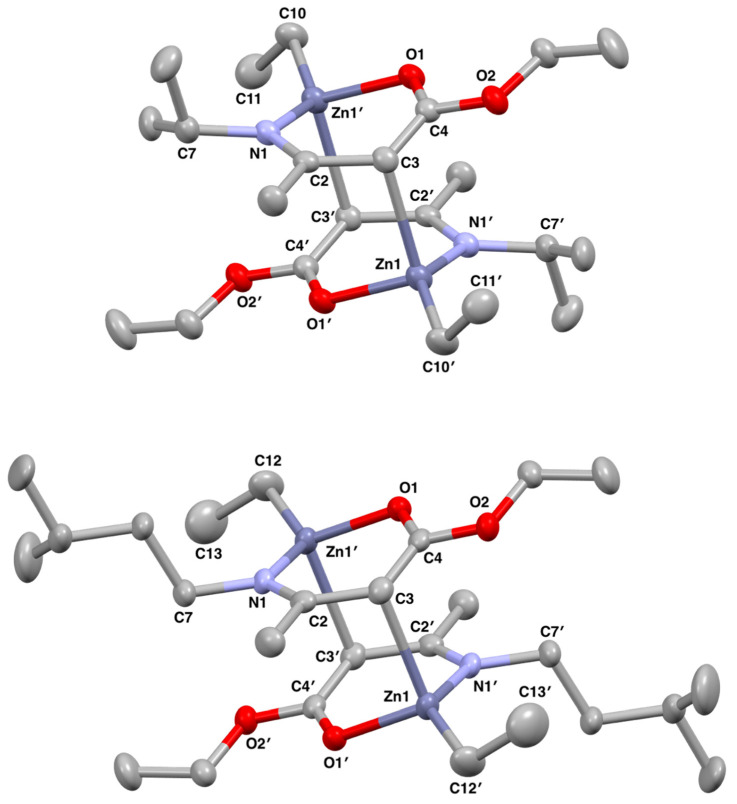
Solid state structures of **1** (**above**) and **2** (**below**) with thermal ellipsoids drawn at 50% probability and with hydrogen atoms omitted for clarity.

The ethyl zinc *β*-amidoenoate complexes **1** and **2**, and the ethyl zinc *β*-ketoiminate complex **3,** were synthesised via 1:1 reactions of diethylzinc with the respective *β*-aminoenoate or *β*-ketoimine in toluene. After reduction of the solvent in vacuo, all three compounds crystallised out of concentrated toluene solutions after being stored at −18 °C for 24 h (**3**) and 48 h (**1**, **2**). Compounds **1**, **2** and **3** were all characterised by ^1^H and ^13^C NMR spectroscopy, as well as single-crystal X-ray diffraction (XRD), which confirmed the structures as dimeric species in the solid state (Figure 2). Peaks observed for the *β*-amidoenoate protons in the ^1^H NMR spectrum of **1** and **2** were all shifted as compared to the free ligands, confirming coordination to the zinc centre, and we also saw triplet–quartet peaks corresponding to the bound ethyl groups appearing as broad signals.

**Figure 3:**
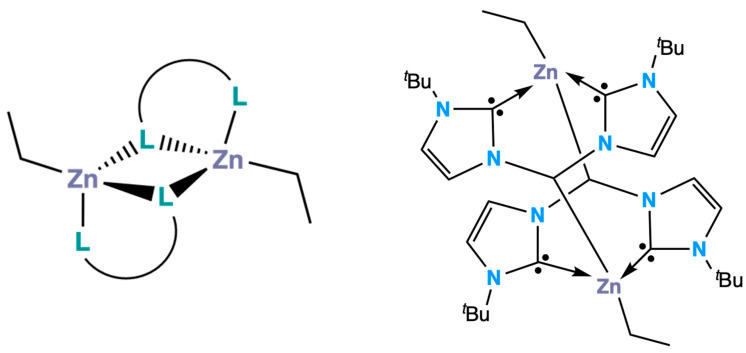
Schematic representation of ethyl zinc complex dimerisation through a [Zn–L]_2_ ring; L represents a donor atom of a bidentate ligand (**left**). Bis(*N*-heterocyclic carbene) zinc ethyl complex reported by Rit et al. exhibiting similar dimerisation to **1** and **2** (**right**).

The ethylzinc *β*-amidoenoates **1** and **2** both crystallised out as centrosymmetric crystals in the triclinic space group *P*$\bar{1}$, each with two zinc centres; one of which was unique and one of which was symmetrically generated about an inversion centre. Both the complexes dimerise analogously, with dative interactions between the zinc atom on one monomeric unit and the methine carbon on the ligand of the second monomeric unit (Figure 3). This dimerisation is uncommon in zinc alkyl complexes, with most dimerisation occurring through a [Zn–L]_2_ (L = donor atom) ring interaction (Figure 3, left) [21,22,23,24]. A bis (N-heterocyclic carbene) zinc ethyl complex previously reported dimerises in a similar fashion to **1** and **2**, though this was due to the unusual deprotonation of the methylene proton of the ligand backbone by diethylzinc, and the formation of a formal Zn–C bond (Figure 3, right) [25].

In both **1** and **2** (Figure 2), two coordination sites around Zn1 are occupied by the coordination of the appropriate bidentate ligand L^1^/L^2^ which bonds through the O1′ and N1′ atoms, and the bonding of L^1^/L^2^ forms a puckered six-membered ZnOC_3_N ring. Zn1 forms a dative interaction with C3 on the symmetry equivalent monomer unit, and as such, **1** and **2** exist as dimers in the solid state with two dative interactions between two monomer units, in which Zn1 adopts a highly distorted tetrahedral geometry. Taking compound **1** as an example, the longest interaction made by Zn1 is the Zn1–C3 interaction between Zn1 on one monomer unit to C3 on the other (2.408(3) Å). The Zn1–C10 bond measures 1.980(3) Å, which is typical of a Zn–C bond. The increased value of the Zn1–C3 distance is highly indicative of a dative interaction between Zn1 and C3. Zn1 also bonds to O1′ and N1′ of the ligand L^1^, with bond lengths of 2.046(2) Å and 2.013(2) Å, respectively (Table 1). The largest bond angle about the Zn atom in **1** is 138.12(13)° for N1′–Zn1–C10, which is over 40° larger than the N1′–Zn1–O1′ angle (93.15(9)°). The large N1′–Zn1–C10 bond angle reduces the steric interaction between the terminal ethyl group on Zn1 and the *^i^*Pr group on N1′. However, the O1′–Zn1–N1′ angle is small due to the steric constraints of the formed ring system. The other angles about Zn1 of O1′–Zn1–C3, O1′–Zn1–C10, N1′–Zn1–C3 and C3–Zn1–C10 have intermediate bond angles of 97.02(9)°, 114.23(11)°, 98.26(9)° and 108.42(12)°, respectively. The deviations from the ideal tetrahedral angle of 109.5° are expected in complexes of this type and result from the inflexibility of the bound chelate ligand and the requirement to reduce the steric interaction between the *^i^*Pr groups and terminal ethyl groups. The trends for bond angles and lengths are analogous for **2** due to both complexes having very similar structures in the solid state.

The bond angles around the four coordinate Zn centres in **1** and **2** both have a range that deviates significantly from the 109.5° that would be observed for a centre with perfect tetrahedral geometry. This is due to the inflexibility of the chelating ligand and the relatively small bite angle resulting from the (OC(OEt)CHC(Me)N(R)) chain, where R is *^i^*Pr in **1** and (CH_2_)_2_*^i^*Pr in **2**. The N1–Zn1–CH_2_CH_3_ bond angle is the largest in both complexes as to reduce the steric conflict in the complexes and place distance between the organic ligand on the N atom and the ethyl group on the Zn atom. The N1′–Zn1–C10 angle in **1** measures 138.12(13)°, compared to the N1–Zn1–C12 bond angle of 133.75(10)° in **2**. The *^i^*Pr group on the N in **1** has a short carbonyl chain and less rotation available about its bonds. This results in the steric bulk of the ligand being in closer proximity to the ethyl group on the Zn, requiring an increased bond angle between them. However, the isopentyl group has a longer carbonyl chain with increased rotation available, which causes a reduction in the steric frustration with the ethyl group on the Zn allowing for a smaller bond angle. This is compensated for by the angle between the O, Zn and C on the ethyl group, which measures 114.23(11)° for the O1′–Zn1–C10 bond angle in **1** and 121.22(9)° for the O1–Zn1–C12 bond angle in **2**.

The Zn centre in both **1** and **2** retains one ethyl group from the diethylzinc while forming two bonds to form a puckered ZnOC_3_N ring from the *β*-aminoenoate ligand and one dative bond to the second monomer unit. Delocalisation in the rings in both complexes is evidenced by the shorter length of the C–O, C–N and C–C bonds within the formed rings when compared to those outside of it. Most bond lengths are slightly shorter in **2**, for example the Zn1–O1 and Zn1–N1 bonds measure 2.0278(14) Å and 1.9964(18) Å, respectively, which compares to 2.046(2) and 2.013(2) Å for the Zn1–O1′ and Zn1–N1′ bonds, respectively, in **1**. However, **2** does have a slightly longer dative bond between the two monomer units, with the Zn1–C3′ measuring 2.452(2) Å compared to the equivalent Zn1–C3 bond measuring 2.408(3) Å in **1**.

The structural parameter τ_4_′ was used to quantify the degree of distortion at the zinc centre. The zinc centres in **1** and **2** adopted highly distorted tetrahedral geometries with τ_4_′ values of 0.69 and 0.71, respectively. It can be argued that the further the τ_4_′ value deviates from the ideal value of 0 (square planar) or 1 (tetrahedral), the more unstable the molecule will be and the more likely it will be that it decomposes at a lower temperature, making it a better contender as a precursor. However, deposition is also dependent on the similarity between the molecular geometry of the precursor and the arrangement of atoms in the desired bulk material, in which case it would be favourable to have a τ_4_′ value closer to ideal values. This is especially true for zinc oxide, where it has been shown that certain geometries of precursors lead to films with certain preferred orientations [26,27]. For zinc oxide, which exists most commonly in the wurtzitic form where both zinc and oxygen atoms adopt tetrahedral geometries, it could be favourable to have precursors with values closer to 1, so as to match the geometries of the precursor to the bulk material.

**Figure 4:**
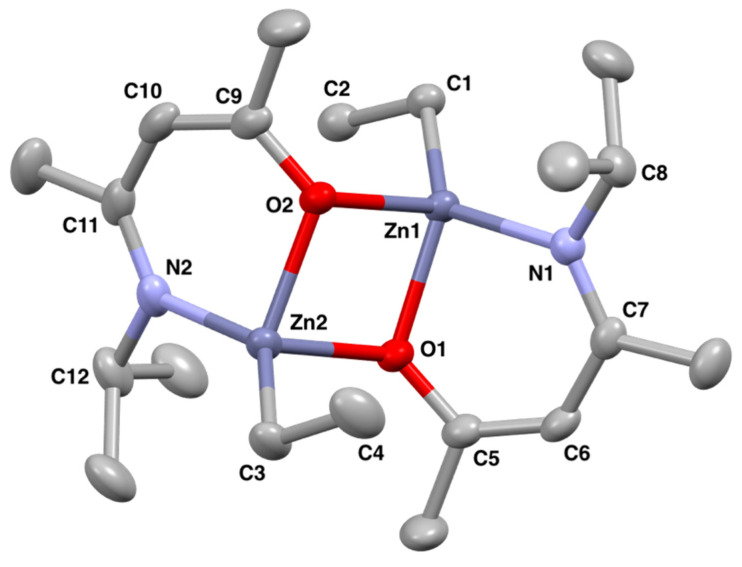
Solid state structure of **3** with thermal ellipsoids drawn at 50% probability and with hydrogen atoms omitted for clarity.

Similarly to **1** and **2**, peaks observed in the ^1^H NMR spectrum of **3** were shifted as compared to the free ligand, indicating that the ligand was coordinated to the zinc centre. The protons corresponding to the bound ethyl group appeared as broad signals, similarly to **1** and **2**. Compound **3** crystallised out of a concentrated toluene solution as a dimeric species in the tetragonal space group *P*4_2_/*n*, with two crystallographically non-equivalent zinc centres (Figure 4). This dimerisation is much more common in zinc complexes, as discussed above. The structural parameter τ_4_′ for **3** was calculated to be 0.73, indicating a highly distorted tetrahedral zinc centre. Each zinc centre is bound to two oxygen atoms in a [Zn-O]_2_ ring structure. All bond lengths including the Zn-O bond lengths are comparable to those in **1** and **2**. The O1–Zn1–N1 bite angle is significantly narrower (by ~10°) than those in **1** and **2**, measuring 83.47(4)°, which is further from the ideal internal tetrahedral angle for a six-membered ring (109.5°). However, the τ_4_′ value for **3** is closer to the ideal tetrahedral geometry of 1 as complexes **1** and **2** have greater than 109.5° bond angles for O1–Zn1–CH_2_CH_3_ and N1–Zn1–CH_2_CH_3_, causing the coordination geometry around the zinc centres to be more distorted.

The 1:1 reaction of HL^3^ with diethylzinc resulted in the formation of **3**, along with the formation of the bis-ligated compound [Zn(L^3^)_2_], even when conducting a cooled dropwise addition. The ratios of **3** to [Zn(L^3^)_2_] from ^1^H NMR was found to be 2:1 for the 1:1 reaction. Repeats of the reaction with 1.6 eq. and 2.5 eq. excess of diethylzinc still resulted in the formation of [Zn(L^3^)_2_], with ratios of 3:1 of **3**:[Zn(L^3^)_2_], which shows the stability of the bis-ligated complex. The bis-ligated complexes [Zn(L^1^)_2_] and [Zn(L^2^)_2_] did not form when conducting these reactions and we believe that this is due to electronic differences in the ligands used. The single-crystal structure of [Zn(L^3^)_2_] has been reported previously, which shows that the compound is monomeric [28].

**Figure 5:**
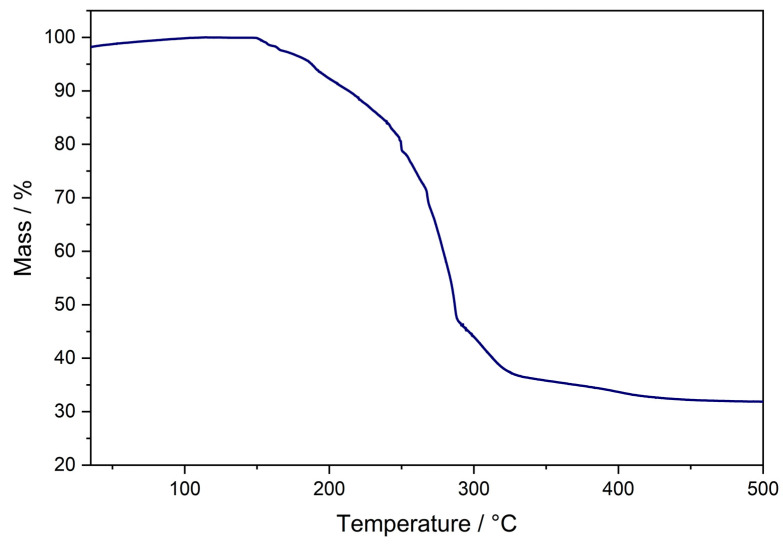
Thermal decomposition profile for the 3:1 mixture of **3**:[Zn(L^3^)_2_].

In **3**, each Zn atom is bound to two O atoms, which is advantageous for deposition of ZnO as there are more pre-formed Zn–O bonds as required in the bulk material. Even though there is a greater atomic percentage of oxygen in **1** and **2**, each Zn atom is only bound to one oxygen atom. As such, thermal gravimetric analysis (TGA) was used to evaluate the efficacy of **3** as a single source precursor to ZnO. Furthermore, TGA analysis was not able to be carried out on **1** and **2** due to their high moisture and air sensitivity. Due to the inseparable mixture obtained from the reaction of diethylzinc with HL^3^, the TGA for this 3:1 mixture of **3**:[Zn(L^3^)_2_] was carried out. The thermal profile shows a two-step decomposition pathway, with an onset decomposition temperature of 150 °C, and a relatively wide decomposition window of ~200 °C (Figure 5).

**Figure 6:**
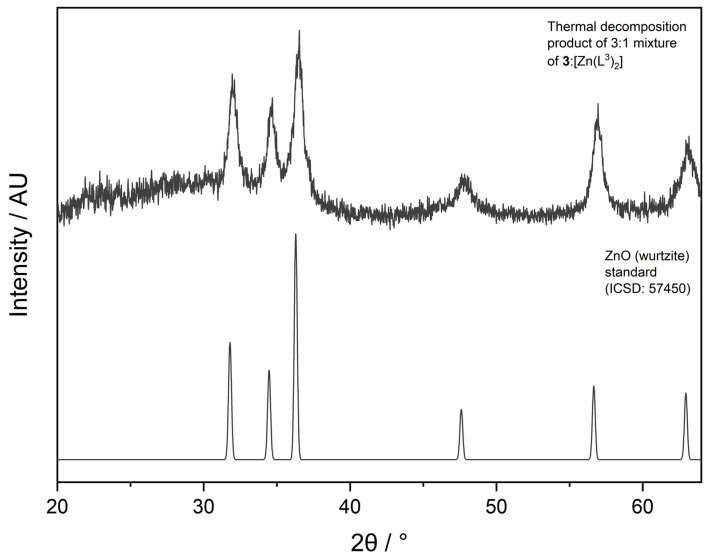
XRD pattern of the thermal decomposition product, ZnO, of the 3:1 mixture of **3**:[Zn(L^3^)_2_].

The TGA profile for a similar bis-ligated zinc *β*-ketoiminate complex, with an *^n^*Bu group on the N atom, showed the onset decomposition to be at 250 °C, with complete decomposition at 400 °C [13]. From this, we may hypothesise that the initial decomposition starting at 150 °C is of compound **3**, with the mass loss at the higher temperature of ~300 °C attributed to the decomposition of [Zn(L^3^)_2_]. The same authors also reported the TGA profile for the bis-ligated complex [Zn(L^1^)_2_], which had an onset decomposition temperature of ~250 °C, with complete decomposition at 400 °C. From the trend seen between **3** and its bis-ligated counterpart, we can infer that **1** and **2** will also have significantly lower decomposition temperatures than their bis-ligated counterparts, perhaps even more so than **3,** as their sensitivity did not allow for TGA analysis to be undertaken. Given that a mixture of 3:1 of **3**:[Zn(L^3^)_2_] was used, it can be calculated that if ZnO was to be formed, a mass% of 32.0% should remain, which is in line with the observed 32.8% final mass% in the TGA, suggesting that this precursor does decompose to ZnO. This was corroborated by XRD analysis of the thermal decomposition product, which confirmed its identity as ZnO (Figure 6).

## 3. Materials and Methods

All preparations were performed under an inert argon atmosphere using standard Schlenk techniques or using an MBraun nitrogen-filled glovebox. All chemicals were obtained from commercial sources. All solvents were obtained from a solvent purification system and stored over molecular sieves. C_6_D_6_ and CDCl_3_ were dried using freeze–pump–thaw cycles and stored over molecular sieves. HL^1^ and HL^3^ were synthesised according to procedures in the literature [14,16].

Single-crystal X-ray diffraction (XRD) data were collected using a SuperNova Atlas (Dual) diffractometer using Cu K*α* radiation of wavelength 1.54184 Å. Suitable crystals were selected and mounted on a nylon loop and the crystal was kept at 150 K during data collection. Nuclear magnetic resonance (NMR) data were recorded in C_6_D_6_ solutions using a Bruker Advance III 500 MHz instrument at ambient temperature. ^1^H and ^13^C{^1^H} NMR assignments were confirmed by ^1^H–^1^H (COSY and NOESY) and ^1^H–^13^C (HSQC and HMBC) experiments where necessary. Thermogravimetric analysis (TGA) measurements were made using a PerkinElmer STA6000 TGA instrument, with a sensitivity of 0.1 mg and using N_2_ as the shield gas. The samples were heated from 30 °C to 500 °C, at a heating rate of 10 °C min^−1^ under the flow of shield gas. XRD patterns were recorded using a Bruker D8 Discover diffractometer.

### 3.1. Synthesis of OC(OEt)CHC(Me)NH(CH_2_)_2_^i^Pr (HL^2^)

Isopentylamine (6.96 cm^3^, 60 mmol) was added dropwise to ethyl acetoacetate (3.97 cm^3^, 30 mmol) dispersed over K-10 montmorillonite clay (20 g) in a 3-necked round-bottom flask fitted with an overhead mechanical stirrer. The reaction slurry initially gave out heat and was stirred at room temperature for 15 h. The product was extracted by washing with dichloromethane (3 × 30 cm^3^) and filtered. The solvent was removed in vacuo to yield a dark orange liquid (yield: 5.43 g, 90%). ^1^H NMR *δ*/ppm (CDCl_3_): 0.89 (6H, d, *J* = 6.7 Hz, (C*H*_3_)_2_CH), 1.22 (3H, *t*, *J* = 7.1 Hz, C*H*_3_CH_2_), 1.43 (2H, q, *J* = 7.1 Hz, CHC*H*_2_), 1.67 (1H, m, (CH_3_)_2_C*H*), 1.89 (3H, s, C*H*_3_C), 3.18 (2H, q, *J* = 6.5 Hz, C*H*_2_NH), 4.05 (2H, q, *J* = 7.1 Hz, CH_3_C*H*_2_), 4.40 (1H, s, C*H*CO), 8.50 (1H, s (broad), N*H*). ^13^C{^1^H} NMR *δ*/ppm (CDCl_3_): 14.8 (*C*H_3_CH_2_), 19.5 (*C*H_3_C), 22.5 ((*C*H_3_)_2_CH), 25.6 ((CH_3_)_2_*C*H), 39.3 (*C*H_2_CH), 41.2 (*C*H_2_NH), 58.3 (CH_3_*C*H_2_O), 81.8 (*C*HCO), 162.1 (q) (*C*NH), 170.8 (q) (*C*O). MS: *m*/*z* [M + H]+: 200.11.

### 3.2. Synthesis of [(Zn(OC(OEt)CHC(Me)N^i^Pr)(Et))_2_] [L^1^ZnEt]_2_ *(**1**)*

A solution of HL^1^ (1.00 g, 5.84 mmol) in toluene (15 cm^3^) was added dropwise to a cooled solution of diethylzinc (1.1 M in toluene) (5.26 cm^3^, 5.84 mmol) in toluene (15 cm^3^). The resulting solution was brought to room temperature and stirred for 24 h. Toluene was partially removed in vacuo and the remaining solution was left at −18 °C for 48 h. The product crystallised out as yellow tinted crystals suitable for analysis via single-crystal XRD (yield: 1.17 g, 75%). ^1^H NMR *δ*/ppm (C_6_D_6_, 500 MHz): 0.32 (4H, br, Zn–C*H*_2_CH_3_), 0.93 (12 H, d, *J* = 6.4 Hz, (C*H*_3_)_2_CH), 1.09 (6H, t, *J* = 7.1 Hz, OCH_2_C*H*_3_), 1.28 (6H, br, Zn–CH_2_C*H*_3_), 1.61 (6H, s, C*H*_3_C), 3.44 (2H, m, (CH_3_)_2_C*H*), 4.03 (4H, q, *J* = 7.1 Hz, OC*H*_2_CH_3_), 4.57 (2H, s, C*H*CO). ^13^C{^1^H} NMR *δ*/ppm (C_6_D_6_, 500 MHz): 4.8 (Zn–CH_2_CH_3_), 11.2 (Zn–CH_2_*C*H_3_), 14.9 (OCH_2_*C*H_3_), 22.2 (*C*H_3_C), 25.6 ((*C*H_3_)_2_CH), 49.4 ((CH_3_)_2_*C*H), 59.5 (O*C*H_2_CH_3_), 79.6 (*C*HCO), 170.3 (q) (*C*N), 172.2 (q) (*C*O). MS: *m*/*z* [M]^+·^: 530.25.

### 3.3. Synthesis of [(Zn(OC(OEt)CHC(Me)N(CH_2_)_2_^i^Pr)(Et))_2_] [L^2^ZnEt]_2_ *(**2**)*

A solution of HL^2^ (1.00 g, 5.02 mmol) in toluene (15 cm^3^) was added dropwise to a cooled solution of diethylzinc (1.1 M in toluene) (4.52 cm^3^, 5.02 mmol) in toluene (15 cm^3^). The resulting solution was brought to room temperature and stirred for 42 h. Toluene was partially removed in vacuo and the remaining solution was left at −18 °C for 48 h. The product crystallised out as yellow tinted crystals suitable for analysis via single-crystal XRD (yield: 0.98 g, 67%). ^1^H NMR *δ*/ppm (C_6_D_6_, 500 MHz): 0.21 (4H, q (br), *J* = 8.1 Hz, Zn–C*H*_2_CH_3_), 0.81 (12H, d, *J* = 6.6 Hz, (C*H*_3_)_2_CH), 1.09 (6H, t, *J* = 7.1 Hz, OCH_2_C*H*_3_), 1.20 (6H, t (br), *J* = 8.1 Hz, Zn–C*H*_2_CH_3_), 1.25 (4H, m, NCH_2_C*H*_2_), 1.41 (4H, m, NC*H*_2_CH_2_), 1.61 (6H, s, C*H*_3_C), 2.98 (2H, m, (CH_3_)_2_C*H*), 4.04 (4H, q, *J* = 7.1 Hz, OC*H*_2_CH_3_), 4.62 (2H, s, C*H*CO). ^13^C{^1^H} NMR *δ*/ppm (C_6_D_6_, 500 MHz): 5.7 (Zn–CH_2_CH_3_), 10.8 (Zn–CH_2_*C*H_3_), 14.9 (OCH_2_*C*H_3_), 21.8 (*C*H_3_C), 22.8 ((*C*H_3_)_2_CH), 26.6 (N*C*H_2_CH_2_), 41.9 (NCH_2_*C*H_2_), 49.2 ((CH_3_)_2_*C*H), 59.6 (O*C*H_2_CH_3_), 79.6 (*C*HCO), 172.6 (q) (*C*N), 172.7 (q) (*C*O).

### 3.4. Synthesis of [(Zn(OC(Me)CHC(Me)N^i^Pr)(Et))_2_] [L^3^ZnEt]_2_ *(**3**)*

A solution of HL^3^ (0.84 g, 6.0 mmol) in toluene (15 cm^3^) was added dropwise to a cooled solution of diethylzinc (1.1 M in toluene) (5.40 cm^3^, 6.0 mmol) in toluene (15 cm^3^). The resulting solution was brought to room temperature and stirred overnight. Toluene was partially removed in vacuo and the remaining solution was left at −18 °C for 24 h after which clear crystals suitable for analysis via single-crystal XRD had formed. ^1^H NMR *δ*/ppm (C_6_D_6_, 500 MHz): 0.67 (2H, br, Zn–C*H*_2_CH_3_), 0.93 (6H, d, *J* = 6.3 Hz, NCH(C*H*_3_)_2_), 1.49 (3H, s, NCC*H*_3_), 1.56 (3H, br, Zn–CH_2_C*H*_3_), 2.01 (3H, s, OCC*H*_3_), 3.38 (1H, hept, *J* = 6.3 Hz, NC*H*(CH_3_)_2_), 4.78 (1H, s, NCC*H*). ^13^C{^1^H} NMR *δ*/ppm (C_6_D_6_, 500 MHz): 1.9 (Zn–CH_2_CH_3_), 12.8 (Zn–CH_2_*C*H_3_), 21.6 (NC(*C*H_3_)), 24.6 (NCH(*C*H_3_)_2_), 27.5 (OC(*C*H_3_)), 50.4 (N*C*H), 98.8 (NC*C*H), 169.6 (*C*N), 179.9 (*C*O).

## 4. Conclusions

We have presented the synthesis of three ethyl zinc complexes as potential SSPs for ZnO thin films. The unusual dimerisation of **1** and **2** led to highly unstable complexes, whilst the more common dimerisation seen in **3** offered greater stability and was therefore the most suitable complex for use as a precursor. As such, TGA was carried out on **3** and its decomposition temperature was significantly lower (~250 °C) than its bis-ligated counterpart [Zn(L^3^)_2_], and this relatively low temperature makes **3** suitable for use as an SSP in CVD processes. Although this reaction yielded a mixture, CVD is usually an in situ process and as such complexes are not isolated. Because the bis-ligated compound [Zn(L^3^)_2_] is an SSP to ZnO in its own right, we do not believe that the inclusion of a small amount of this would hinder the growth of ZnO when undertaking the deposition of **3** to form ZnO.

The usage of chelating *β*-amidoenoate and *β*-ketoiminate ligands along with one ethyl group remaining from the diethylzinc has given rise to a set of heteroleptic complexes with properties intermediate to diethylzinc and bis-ligated zinc *β*-amidoenoates and *β*-ketoiminates. They are not pyrophoric due to the chelate ligand, but they are still highly sensitive to air and moisture due to the remaining ethyl group, and are therefore promising as SSPs for ZnO, as evidenced by the TGA data of **3** and its thermal decomposition product confirmed as ZnO by XRD analysis.

## Figures and Tables

**Table 1:** Selected bond lengths (Å) and bond angles (°) for compounds **1**, **2** and **3**.

Bond Lengths/Å	1	2	3
Zn1-O1	2.046(2)	2.0278(14)	2.0233(10)
Zn1-O2	-	-	2.1329(10)
Zn1-N1	2.013(2)	1.9964(18)	2.0262(11)
Zn1-*C*H_2_CH_3_	1.980(3)	1.962(2)	1.9806(14)
O1-C	1.252(4)	1.254(3)	1.3141(17)
**Bond Angles/°**			
O1-Zn1-N1	93.15(9)	92.99(7)	83.47(4)
O1-Zn1-CH_2_CH_3_	114.23(11)	121.22(9)	123.96(5)
N1-Zn1-CH_2_CH_3_	138.12(13)	133.75(10)	130.03(5)

## Data Availability

The data presented in this study are available on request from the corresponding author.

## Sample Availability

Samples of the compounds are not available from the authors.
